# Interaction of breathing pattern and posture on abdominal muscle activation and intra-abdominal pressure in healthy individuals: a comparative cross-sectional study

**DOI:** 10.1038/s41598-023-37629-5

**Published:** 2023-07-13

**Authors:** Masashi Kawabata, Norihiro Shima

**Affiliations:** 1 Department of Rehabilitation, School of Allied Health Sciences, Kitasato University, Sagamihara, Kanagawa Japan; 2 School of Sport and Health Science, Tokai Gakuen University, 21-233, Nishinohora, Ukigai, Miyoshi, Aichi 470-0207 Japan

**Keywords:** Physiology, Health care

## Abstract

We aimed to assess the effects of interaction between several breathing patterns and postures on abdominal muscle activation and intra-abdominal pressure (IAP). This comparative cross-sectional study enrolled fourteen healthy university students majoring in sports science and/or physical education. They performed four active breathing tasks: quiet nasal breathing (Q-Bre), nasal deep breathing (Deep-Bre), completely forced expiration (Forced-Expi), and exertional nasal inhalation with abdominal muscles with isometric contraction (Exertion-Inspi) in the elbow-toe plank and supine postures. Breathing volume; IAP; and transverse abdominis-internal oblique muscle (TrA-IO) and external oblique muscle (EO) activities were recorded. Abdominal muscle activity and IAP significantly interacted with breathing pattern and postures during the expiratory phase (*p* < 0.05). In the inspiratory phase, TrA-IO activity was significantly affected by breathing pattern and EO activity with posture (*p* < 0.05). TrA-IO activity significantly increased during Forced-Expi in the supine posture (47.6% of the maximum voluntary contraction) and Exertion-Inspi in the elbow-toe posture (35.7%), whereas no differences were found during Deep-Bre or Q-Bre (< 20%). EO activity increased in the elbow-toe posture (22.5–30.6%) compared with that in the supine posture (< 5%) during all breathing tasks. IAP values were low during all tasks (< 15%) except for Forced-Expi (24.9%). Abdominal muscle activation and IAP interacted with the breathing pattern and posture.

## Introduction

Breathing exercises, such as maximum expiration training^1^, respiratory resistance training^2^, inspiratory muscle training^3–5^, or Pilates^3^, are well-known for enhancing core stability and physical fitness. These exercises may be effective in preventing low back pain in athletes^6,7^ or as therapeutic exercises in patients with low back pain with a positive lumbar instability test^2^. In addition, inspiratory muscle training has been found to improve dynamic balance in healthy older adults, resulting in improved walking speed and inspiratory muscle function^4,5^. Thus, breathing exercises could improve physical function, human behavior, and quality of life, which are not merely for improving breathing function^3^.

Breathing exercises are often combined with the motion of the limbs or trunk, such as with the plank, elbow-toe, and postural stability exercises. Combining exercises could lead to a synergistic effect because the respiratory muscles are used for both respiration and postural control^8–10^. There is a trade-off between the task demand such as posture or limb movements and respiratory demand of the respiratory muscles (diaphragm, internal oblique, and transverse abdominal muscle), which are called the local muscles^9,11–15^. For instance, during heavy weight-lifting, spinal stability is the priority, at the expense of a steady breathing pattern^11,13^. However, as respiratory demand is increased, such as in certain exercises or in cases of respiratory disease, the contribution of the transverse abdominal muscle and diaphragm to spinal stability could be compromised^9,12^. These basic studies have led to the principle of performing exercise in conjunction with the correct posture and breathing for injury prevention and enhancing physical function.

Similarly, some clinical practice studies have demonstrated the effect of exercises combined with breathing. Abdominal draw-in lumbar stabilization exercises with respiratory resistance resulted in decreased low back pain and dysfunctions and increased muscle thickness in contraction, contraction rate, and pulmonary function^2^. Another clinical practice study has shown that the activity of the abdominal muscles increased significantly when combined with maximum expiration compared with resting expiration during side-bridge exercises^16^. In addition, inspiratory muscle training combined with the Pilates methods was found to provide an enhancement in pulmonary function and physical conditioning in older patients^3^. However, basic and clinical practice studies have not bridged between several breathing patterns and postures on abdominal muscle activation and intra-abdominal pressure (IAP) caused by these muscles co-contraction. Clinicians should elucidate how much IAP would increase by exercises^17,18^ and consider whether exercises allow individuals to sustain exercise without cardiorespiratory burden^11,13^. Thus, understanding the effects of breathing pattern and posture from a physiologic standpoint is necessary for effective exercise instruction. This study aimed to examine the effects of interaction between several breathing patterns and postures on abdominal muscle activation and IAP.

## Methods

### Participants

Fourteen healthy men (mean age, 22.7 years [standard deviation (SD), 5.0 years], mean height, 172.1 cm [4.2 cm], and mean weight, 73.3 kg [13.4 kg]) volunteered to participate in this comparative cross-sectional study. The participants were university students majoring in physical education. The study sample comprised four football players, two rugby players, two long-distance runners, a sprinter, a tennis player, a baseball player, a volleyball player, a handball player, and a soccer player. The participants had no neuromuscular, orthopedic, or respiratory abnormalities.

All procedures involving human participants were performed in accordance with the ethical standards of the Institutional and/or National Research Committee and the 1964 Helsinki declaration and its later amendments or comparable ethical standards. Written informed consent was obtained from all participants. The protocol was approved by the Ethics Review Board of Tokai Gakuen University (# 24-19).

### Task

**Figure 1:**
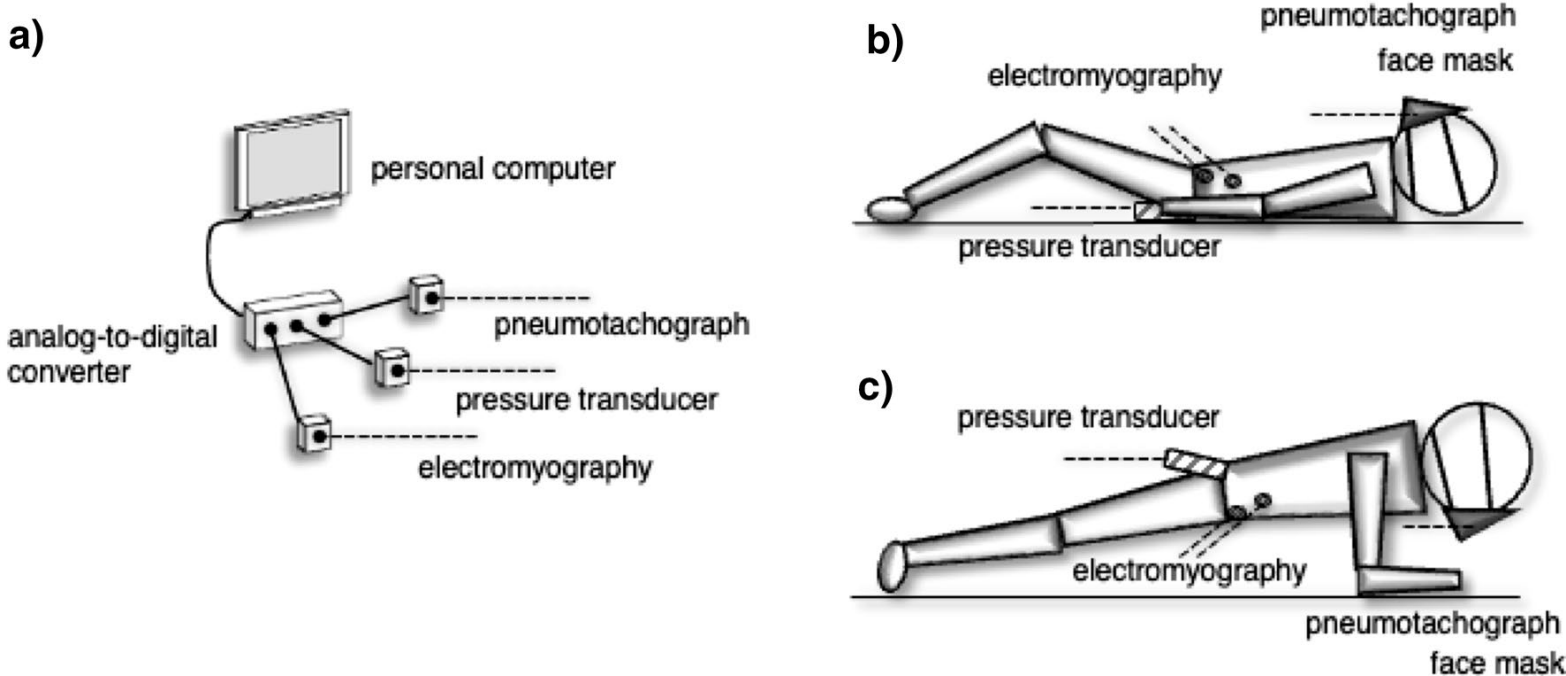
A schematic diagram of the experimental setup (**a**) for the active breathing task in the supine (**b**) and elbow-toe plank (**c**) postures. Intra-abdominal pressure data are collected by a pressure transducer placed intra-rectally. Airflow data are measured by a pneumotachograph attached to a face mask covering the nose and mouth. Abdominal muscle activity is measured during the task using electromyography. All participants perform the active breathing exercise at least three times. All elbow-toe tasks are performed with a straight trunk and lower extremities for 30 s.

The participants performed four active breathing tasks: quiet breathing (Q-Bre), deep breathing (Deep-Bre), pursed-lips forced expiration (Forced-Expi), and exertional nasal inhalation with expanding the thorax not to abdominal wall expansion (Exertion-Inspi). Specifically, Q-Bre was normal nasal tidal breathing (as a control). Deep-Bre was a complete nasal inhalation with submaximal expansion of the abdominal wall and relaxed expiration through the mouth. Forced-Expi was a completely forced exhale with pursed lips until the abdominal wall was hollowed. Exertion-Inspi was a complete nasal inhalation, sufficient to expand the thorax while maintaining the abdominal muscles in isometric contraction, not to abdominal wall expansion. The respiratory rhythm was applied by examiner cueing. The participants practiced repeatedly during a lesson from breathing exercise instructors (T.K. and J.U.). The four breathing tasks were performed in a random order in the supine (as a control posture) and elbow-toe postures for 30 s. Regarding the elbow-toe posture, the participants performed a prone plank posture on the floor, maintaining a straight trunk and lower extremities, with only their toes and forearms touching the floor (Fig. 1). They rested for 3 min between the tasks to eliminate the influence of fatigue.

### IAP, breathing, and muscle activity measurements

Respiratory and IAP measurements were performed as previously described^19,20^. Briefly, IAP was measured using a pressure transducer (MPC-500, Millar Instruments, Inc., Houston, TX, USA) placed intra-rectally^19–21^. Before the actual measurements were taken, the maximum IAP was obtained based on the maximum voluntary pressurizations produced during Valsalva maneuvers performed in a standing posture (_max_IAP). The highest value among three trials was used as the maximum. The IAP during the task was normalized using _max_IAP (%IAP). The breathing volume of all the tasks was measured with a pneumotachograph (FM-200, Arco System, Inc., Kashiwa, Japan) using a face mask covering the nose and mouth (7400, Hans Rudolph Inc., Wyandotte, MO, USA). Respiratory volume was calibrated using a syringe calibrated at 3 L (763722, Sensor Medics Corp., Yorba Linda, CA, USA).

Surface electromyogram (EMG) measurements were conducted as previously described^22^. Preparing for the EMG, the surface of the skin was cleaned with alcohol and rubbed with sandpaper. Surface bipolar electrodes (Ag–AgCl, 6-mm contact diameter, 1.5-cm inter-electrode space) were placed on each of the muscles using Kinesio tape. Before measurements and throughout the testing period, all EMG signals were monitored and checked using a real-time oscilloscope display. EMG signals from the right transverse abdominis-internal oblique muscle (TrA-IO) and right external oblique muscle (EO) were recorded during the tasks. Electrodes for the TrA-IO were placed 2 cm inferomedially to the anterior superior iliac spine^23^ at an angle following the inguinal ligament. The activity of the TrA-IO could be best assessed from this surface location^24,25^. Electrodes for the EO were placed at the junction of a line drawn from the umbilicus to the anterior axillary line at an oblique angle following the muscle fibers. A reference electrode was placed on the right iliac crest. The EMG signals were amplified differentially using an AC amplifier (input impedance 5 MΩ, gain 1000–2000×, common-mode rejection ratio > 60 dB). Moreover, band-pass filtering was set at both low (time constant of 0.03 s) and high (2 kHz) cut-off filters (AB-620G, Nihon Koden, Japan). Before performing the actual tasks, all participants completed three consecutive trials of maximum voluntary contraction (MVC) of the isometric muscles^21^. MVC was performed to sustain the posture against the external force applied in the supine (for TrA-IO) and side-lying (for EO). The participants kept the area beyond their iliac crest hanging over the examination table in the supine or side-lying posture, with their pelvis and lower leg held in place by two examiners. Thereafter, another examiner applied the maximal resistance to the participant’s upper trunk with the hands using the whole body weight. The participants horizontally kept their trunk position against resistance, trying to hold the maximum force for approximately 5 s. The highest root mean square of EMG amplitude of each muscle during the three MVC trials (or Valsalva maneuver tests as mentioned below) was adopted as the MVC value used for subsequent statistical analysis (100% MVC). The root mean square of EMG data of the MVC trial was calculated for 100 ms (50 ms before and after the peak value). Moreover, Valsalva maneuver test was performed in the standing position to develop the maximal IAP (100% IAP)^19,20^. Valsalva maneuver test comprised a complete nasal inhalation followed by holding breath with maximal voluntary pressurization to the abdominal compartment as forcefully as possible.

**Figure 2:**
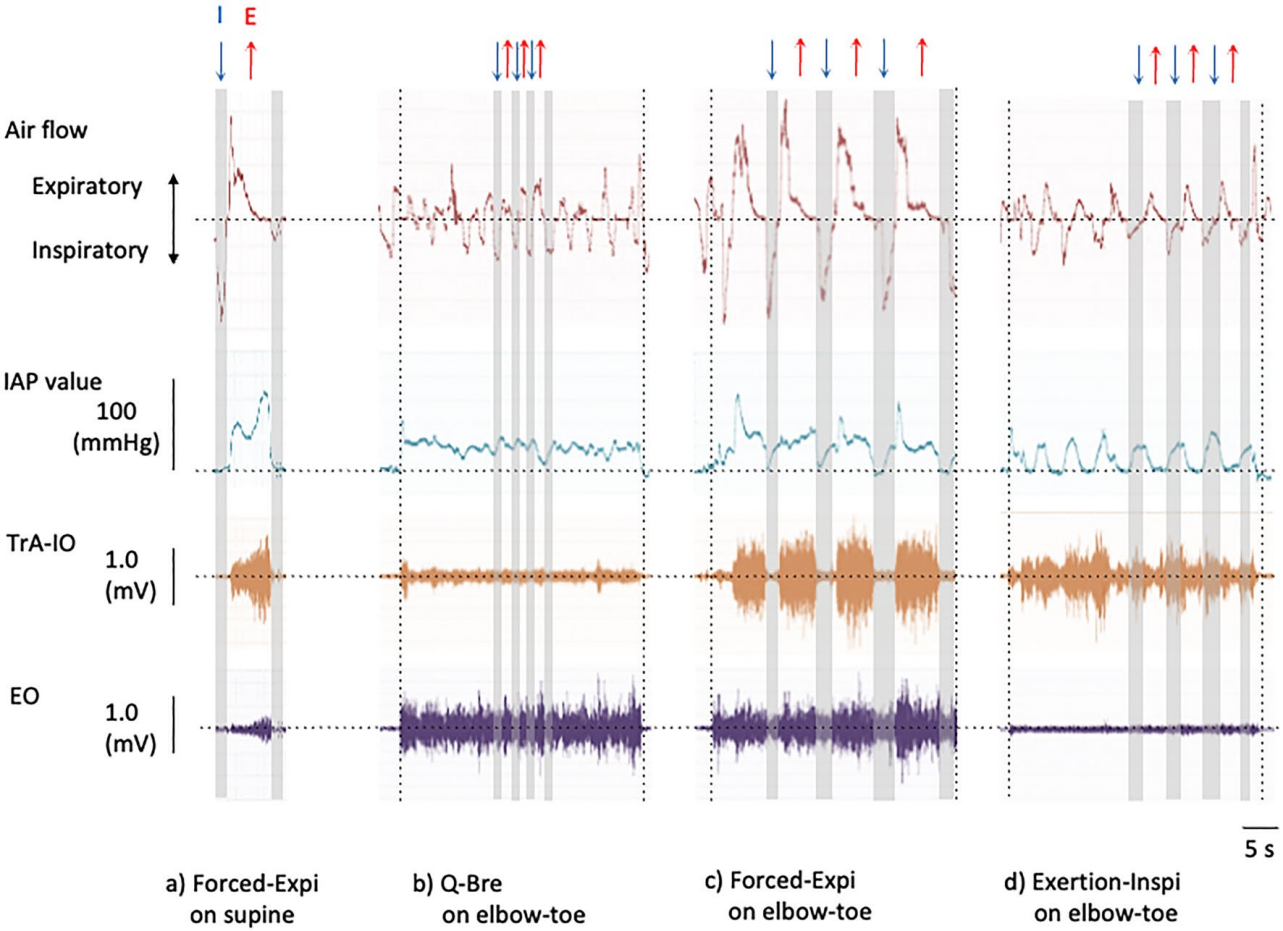
Representative data of air flow, IAP, and electromyography during the active breathing task (**a**–**d**), with the inspiratory (↓) and expiratory phase (↑). The vertical dotted line indicates the elbow-toe posture interval. *I* inspiratory phase (↓), *E* expiratory phase (↑), *IAP* intra-abdominal pressure, *TrA-IO* transverse abdominal and internal oblique muscle, *EO* external oblique muscle, *Q-Bre* quiet nasal breathing, *Forced-Expi* completely forced expiration with pursed-lips until the abdominal wall hollowed, *Exertion-Inspi* exertional nasal inhalation with abdominal muscle isometric contraction to prevent abdominal wall expansion.

IAP and airflow and EMG data were simultaneously recorded on a computer (chart 5.3, AD Instruments, Sydney, Australia) using an analog-to-digital converter (Power-lab 8sp, AD Instruments, Sydney, Australia) at a sampling rate of 2 kHz. Representative data are shown in Fig. 2. For each parameter, values were averaged from a series of three trials for each set of phases (inspiratory and expiratory).

### Statistical analysis

All data are expressed as mean ± standard error of mean. A two-factor (breathing and posture) repeated-measures analysis of variance was performed to assess statistical significance of the effects of interaction. Based on the model of Mauchly’s test, if the assumption of sphericity was not met, the Greenhouse–Geisser correction was applied. Then, Bonferroni post hoc testing was used for pairwise comparisons. All statistical analyses were performed using SPSS software, version 22 (SPSS Inc., Chicago, IL, USA). A *p*-value < 0.05 was considered statistically significant. In addition, the magnitude of changes in the values from the reference value (Q-Bre in the supine posture) was expressed as the effect size. Each outcome was classified as a small (0.2), moderate (0.5), or large (≥ 0.8) effect^26^. Finally, the muscle activity ratio between the local and global muscles was expressed as a relative value (TrA-IO/EO).

## Results

Respiratory volume was remarkably different between breathing patterns and posture during both the inspiratory and expiratory phases (Table 1). Forced-Expi was found to result in the largest respiratory volume by a notable margin, followed by Deep-Bre, in which the respiratory volume was higher than that in Q-Bre. No differences in volume were found between Exertion-Inspi and Q-Bre.

**Table 1:** Mean ± Standard error of mean (95% confidence interval [CI]) respiratory volume values (liter).

	Posture	Breathing task				F value (*p* value)	*p* < 0.05 by Bonferroni testing
		(a) Q-Bre	(b) Deep-Bre	(c) Forced-Expi	(d) Exertion-Inspi		
Inspiratory phase	Supine	0.75 ± 0.07 (0.60–0.90)	1.51 ± 0.12 (1.25–1.76)	2.57 ± 0.20 (2.13–3.01)	0.93 ± 0.07 (0.79–1.08)	61.64 (0.001*)	c versus a, b, d b versus a, d
	Elbow-toe	0.67 ± 0.11 (0.44–0.91)	1.26 ± 0.12 (0.99–1.53)	1.81 ± 0.19 (1.40–2.22)	1.12 ± 0.11 (0.88–1.37)		c versus a, b, d b versus a
Posture						4.05 (0.066)	
Expiratory phase	Supine	0.69 ± 0.08 (0.53–0.85)	1.44 ± 0.12 (1.17–1.71)	3.33 ± 0.27 (2.76–3.90)	0.98 ± 0.09 (0.79–1.17)	69.45 (0.001*)	c versus a, b, d b versus a
	Elbow-toe	0.55 ± 0.06 (0.41–0.69)	1.34 ± 0.12 (1.09–1.60)	2.31 ± 0.23 (1.81–2.81)	1.11 ± 0.14 (0.82–1.41)		c versus a, b, d b versus a
Posture						7.72 (0.016*)	

*Q-Bre* quiet nasal breathing, *Deep-Bre* nasal deep breathing until abdominal wall expansion, *Forced-Expi* completely forced expiration with pursed-lips until the abdominal wall hollowed, *Exertion-Inspi* exertional nasal inhalation with abdominal muscle isometric contraction to prevent abdominal wall expansion.

*p < 0.05.

TrA-IO and EO activity including IAP only had a remarkable effect on the breathing pattern and posture during the expiratory phase (Table 2). In the inspiratory phase, the breathing task remarkably affected TrA-IO activity and IAP, whereas the posture affected EO activity and IAP (Table 2).

**Table 2:** Results of a two-factor repeated-measures analysis of variance of muscle activity and intra-abdominal pressure (IAP) development.

	Breathing task		Posture		Posture × breathing task	
	F value	*p* value	F value	*p* value	F value	*p* value
Inspiratory phase						
TrA-IO	10.878	0.002*	3.906	0.070	2.053	0.164
EO	0.286	0.835	74.904	0.001*	2.110	0.146
IAP	9.459	0.001*	54.695	0.001*	2.219	0.101
Expiratory phase						
TrA-IO	12.488	0.001*	0.388	0.544	6.222	0.009*
EO	6.522	0.014*	76.044	0.001*	4.258	0.011*
IAP	19.850	0.001*	5.104	0.042*	34.574	0.001*

*TrA-IO* transverse abdominal and internal oblique muscle, *EO* external oblique muscle.

*p < 0.05.

**Figure 3:**
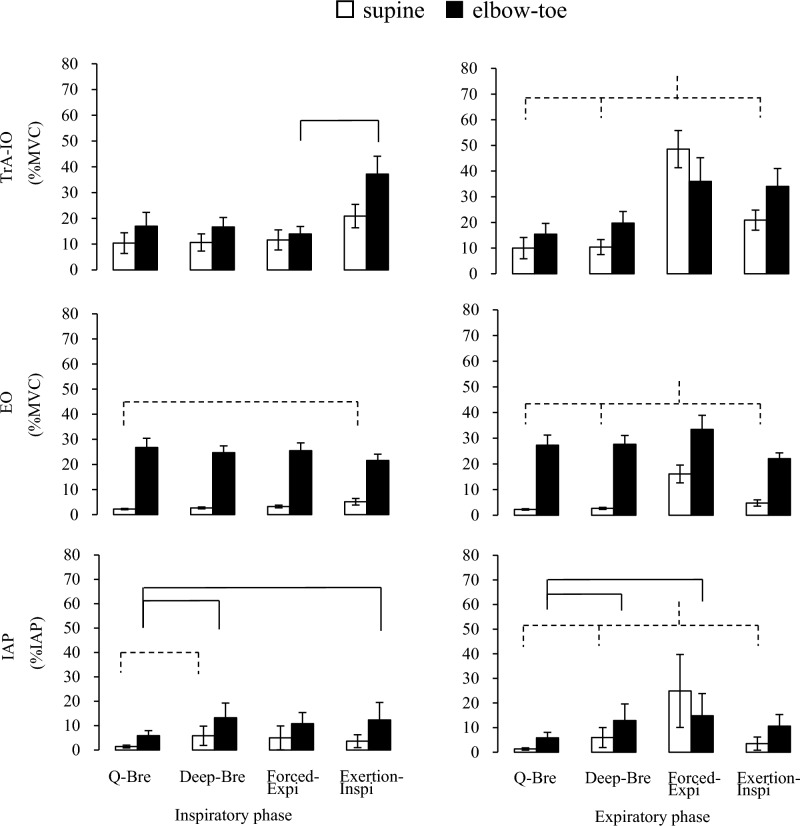
Mean and standard error of the mean (SEM) values for muscle activity and IAP. Dashed line (supine posture) and continuous line (elbow-toe posture): *p* < 0.05 (using Bonferroni testing). *IAP* intra-abdominal pressure, *EO* external oblique muscle, *MVC* maximum voluntary contraction, *TrA-IO* transverse abdominal and internal oblique muscle, *Q-Bre* quiet nasal breathing, *Deep-Bre* nasal deep breathing until abdominal wall expansion, *Forced-Expi* completely forced expiration with pursed-lips until the abdominal wall is hollowed, *Exertion-Inspi* exertional nasal inhalation with abdominal muscle isometric contraction to prevent abdominal wall expansion.

The greatest TrA-IO activity was observed in Forced-Expi at the expiratory phase (Fig. 3). The next greatest activity exceeded 30% of the MVC in Exertion-Inspi, in both inspiratory and expiratory phases in the elbow-toe posture (Table 3). No differences were found below 20% of the MVC in Deep-Bre or Q-Bre, regardless of the posture, and in the TrA-IO during Exertion-Inspi in the supine posture (Fig. 3, Table 3).

**Table 3:** Effect size (Cohen’s d) referenced by quiet nasal breathing (Q-Bre) in the supine posture.

	Posture	Breathing task			
		Q-Bre	Deep-Bre	Forced-Expi	Exertion-Inspi
Inspiratory phase					
TrA-IO	Supine	*ref*	0.02	0.08	0.69^†^
	Elbow-toe	0.40	0.39	0.17	1.57*
EO	Supine	*ref*	0.49	1.07*	3.07*
	Elbow-toe	21.84*	21.14*	21.40*	20.12*
IAP	Supine	*ref*	7.49*	6.04*	3.69*
	Elbow-toe	7.55*	20.01*	15.92*	18.57*
Expiratory phase					
TrA-IO	Supine	*ref*	0.03	2.26*	0.69^†^
	Elbow-toe	0.31	0.57^†^	1.34*	1.42*
EO	Supine	*ref*	0.44	12.82*	2.51*
	Elbow-toe	20.81*	22.34*	26.71*	19.60*
IAP	Supine	*ref*	10.53*	53.43*	4.94*
	Elbow-toe	10.33*	26.20*	30.60*	21.07*

*ref* reference value, *IAP* intra-abdominal pressure, *TrA-IO* transverse abdominal and internal oblique muscle, *EO* external oblique muscle, *Q-Bre* quiet nasal breathing, *Deep* nasal deep breathing until abdominal wall expansion, *Forced-Expi* completely forced expiration with pursed-lips until the abdominal wall hollowed, *Exertion-Inspi* exertional nasal inhalation with abdominal muscle isometric contraction to prevent abdominal wall expansion.

*Large effect (*d* > 0.8).

^†^Moderate effect (*d* > 0.5).

EO activity in the elbow-toe posture induced 22.5–30.6% of the MVC regardless of the type of breathing pattern (Fig. 3, Table 3). In contrast, EO activity in the supine posture resulted in < 5% of the MVC, except for the remarkably increased activity in Forced-Expi at the expiratory phase (15.8% of the MVC) (Fig. 3).

The relative values (TrA-IO/EO) were > 1.0 in all breathing tasks in the supine posture (Table 4). In the elbow-toe posture, these values were only > 1.0 in Exertion-Inspi at both the inspiratory and expiratory phases and in Forced-Expi at the expiratory phase. In contrast, these values were < 1.0 in the other breathing tasks due to the larger increase in activity contribution with EO compared with that with TrA-IO.

**Table 4:** Relative value of TrA-IO and EO activities.

		Posture	Breathing task			
			Q-Bre	Deep-Bre	Forced-Expi	Exertion-Inspi
Inspiratory phase						
TrA-IO/EO	(ratio)	Supine	4.96	4.11	3.68	4.12
		Elbow-toe	0.69	0.71	0.56	1.59*
Expiratory phase						
TrA-IO/EO	(ratio)	Supine	4.66	3.98	3.01	4.46
		Elbow-toe	0.62	0.75	1.06*	1.46*

*TrA-IO* transverse abdominal and internal oblique muscle, *EO* external oblique muscle, *Q-Bre* quiet nasal breathing, *Deep* nasal deep breathing until abdominal wall expansion, *Forced-Expi* completely forced expiration with pursed-lips until the abdominal wall hollowed, *Exertion-Inspi* exertional nasal inhalation with abdominal muscle isometric contraction to prevent abdominal wall expansion.

*Relative value > 1.0 in elbow-toe task.

The maximum IAP during the Valsalva maneuvers was 196.9 mmHg (SD, 59.5; range, 67.1–295.0). The relative IAP value was substantially lower in all tasks, at < 15% of the IAP (Fig. 3, Table 3). Only Forced-Expi at the expiratory phase was notably increased in the supine posture (24.9% of the IAP).

## Discussion

In this study, we found novel findings showing an effect of interaction between several combinations of breathing patterns and postures on abdominal muscle activities based on EMG and IAP, while observing respiratory volume. We found that TrA-IO and EO activity including IAP only interacted with breathing pattern and posture during the expiratory phase. Additionally, in the inspiratory phase, TrA-IO activity and IAP were remarkably affected by breathing pattern, while EO activity and IAP were affected by postural tasks. Thus, our results may fill the gap between basic and clinical practice regarding the importance of combining posture exercises with breathing tasks.

The greatest TrA-IO activity was observed in the Forced-Expi breathing pattern at the expiratory phase in the supine posture (47.6% of MVC). Even in the supine posture, the TrA-IO activity level in Forced-Expi breathing was still relatively higher than that in some of the plank exercises without breathing tasks reported in previous studies^27,28^. TrA-IO activity was primarily affected by Forced-Expi, as evidenced by the greatest IAP development, which induced the greatest respiratory volume approximately 4–5 times, compared with that in Q-Bre (Table 1). In the qualitative observation of IAP dynamics and TrA-IO activity, Forced-Expi induced primary positive pressure in the expiratory phase, corresponding with the phasic activity of the TrA-IO after negative pressure in the inspiratory phase (Fig. 2c). According to observations of ultrasonographic visual change, the mean thickness of the IO muscle significantly increases at end-expiration phases^29^. Furthermore, TrA/IO thickness increases by approximately 1 mm, a considerable increase, after the breathing exercise of maximum expiration with the maximal abdominal contraction maneuver^1^. Forced expiration corresponding with the positive pressure would promote concentric activity of the TrA-IO. However, we must consider the risk–benefit ratio between cardiorespiratory burden and exercise intensity. The greatest IAP value in the Forced-Expi (24.9% of IAP) was moderate and comparable with the result of 45% maximal lifting effort during isometric weight-lifting found in a previous study^19^. If a few breathing trials did not show any issues, but were repeated several times, it was still necessary to consider the cardiorespiratory burden.

The second greatest TrA-IO activity value was observed in the Exertion-Inspi pattern throughout the inspiratory and expiratory phases in the elbow-toe posture (approximately 30–40% of MVC). Despite this finding, no statistical differences in respiratory volume were found between Exertion-Inspi and Q-Bre (Table 1). The TrA-IO activity was triggered by the inspiratory phase rather than by the expiratory phase, contrary to the pattern observed in Forced-Expi (Fig. 2d). The Exertion-Inspi method resembles the Pilates methods^30^, which was a complete nasal inhalation while maintaining the abdominal muscles in isometric contraction, not to abdominal wall expansion. Although the respiratory volume was not highly increased, to maintain the isometric contraction, the TrA-IO activity required to be balanced against the increasing pressure produced by the descent of the diaphragm in inhalation^31,32^. Thus, endurance-related training is recommended for the sustenance of TrA/IO activity without being affected by the breathing event, as TrA/IO activity plays a role in respiration and the postural control effect^8–10^. Moreover, it would improve the risk–benefit ratio, because the IAP value in Exertion-Inspi was smaller (12.4% of IAP) than that in Forced-Expi. This low value allows for sustaining exercise without cardiorespiratory burden^19^. Regarding exercise instruction, due to the small respiratory volume in Exertion-Inspi, the instructor emphasized the need for isometric contraction of the TrA-IO.

Compared with the Q-Bre task, there was no increase in the activity of the TrA-IO in the Deep-Bre. TrA-IO activity in Deep-Bre in the elbow-toe posture was only approximately 20% of the MVC, which was the same as that of some of the plank exercises without breathing in previous studies^27,28^. Even if we focus on the large inspiration pattern, which induced a 2.0–2.4-fold respiratory volume in Deep-Bre compared with that in Q-Bre, the relaxed-deep breathing (Deep-Bre) resulted in insufficient TrA-IO activity. In this breathing pattern, the abdominal muscles only expanded passively, contrary to what was observed in Exertion-Inspi. Although the IAP value in Deep-Bre was low, the elasticity of the passive expansion of abdominal muscles nullified the IAP produced by the descent of the diaphragm during inhalation^31,32^. Thus, the Deep-Bre pattern did not affect TrA-IO activity and IAP dynamics and only promoted the passive expansion of the abdominal muscles, even in the elbow-toe posture.

EO activity was affected by the posture tasks. The activity was 3–6 times larger in the elbow-toe posture than that in the supine posture, regardless of the type of breathing pattern. Although this result is in accordance with the results of previous studies^27,28^, it is impossible to compare them directly due to differences in MVC trials and lack of supine posture data in the previous studies^27,28^. Imai et al.^27^ and Okubo et al.^28^ found that EO activity increased in several additional postural tasks in addition to the elbow-toe posture, rather than in the local muscles. These results in combination with ours suggest that EO activity contributes more to posture tasks than to breathing-related tasks.

We found a unique result regarding the relative values of TrA-IO and EO activity. The TrA-IO/EO ratio of < 1.0 in the elbow-toe posture indicated that the contribution of EO activity was higher than that of TrA-IO activity. Conversely, a ratio > 1.0 in Exertion-Inspi in the inspiratory-expiratory phase and in Forced-Expi in the expiratory phase indicated that the TrA-IO was also a contributor, together with the EO. Performing the dual task of posture and breathing may be useful for activating all the abdominal muscles. The dual task is also likely to have an effect on muscle strength and physical performance. Additional research is required for determining the training effect on physical performance and synergistic effects of muscle function.

There are several limitations to this study. First, the participants were university students majoring in physical education who were not accustomed to routine practice of the breathing methods used, such as the Pilates method. Abdominal draw-in lumbar stabilization exercises in the resistance respiratory group were received three times per week for 4 weeks, resulting in reduced pain and abdominal dysfunction compared with the control middle-aged group^2^. On the other hand, we could not evaluate the prospective study about the trainability or sustained effects of training according to each sport’s characteristics in this study. Second, we used only surface EMG rather than fine-wire EMG. Fine-wire EMG could have been used to clarify each muscle activity with less cross-talk effect^27,28^. However the muscle activity of the TrA-IO and EO significantly differed among several breathing patterns and postural tasks in this study. Thus, our goal might have been achieved. Lastly, we could not directly assess the activity of the diaphragm due to technical reasons, such as using fine-wire EMG as an invasive method for the diaphragm^9,12^. Instead, we considered that measuring IAP value played a role in estimating the activity of the diaphragm including the abdominal muscles.

## Conclusion

The TrA-IO, EO, and IAP were remarkably affected by breathing pattern and posture in the expiratory phase. Meanwhile, in the inspiratory phase, TrA-IO activity and IAP affect the breathing pattern, whereas EO activity had a relationship with the posture. For appropriate exercise load on the abdominal muscles, it is recommended to combine postural tasks with forced breathing, not relaxed-deep breathing.

## Data Availability

The data presented in this study are available on a reasonable request from the corresponding author.
